# Ideational Slippage in Middle-Aged and Older Adults: A Preliminary Study

**DOI:** 10.3390/ijerph21060656

**Published:** 2024-05-22

**Authors:** Eamonn P. Arble, Steven W. Steinert, Sneha Shankar, Alex Cerjanic, Bradley P. Sutton, Ana M. Daugherty

**Affiliations:** 1 Department of Psychology, Eastern Michigan University, Ypsilanti, MI 48197, USA; stevenwsteinert@gmail.com (S.W.S.); sshankar@emich.edu (S.S.); 2 Beckman Institute for Advanced Science and Technology, University of Illinois Urbana-Champaign, Urbana, IL 61801, USA; acerjanic@mgh.harvard.edu (A.C.); bsutton@illinois.edu (B.P.S.); 3 Department of Bioengineering, University of Illinois Urbana-Champaign, Urbana, IL 61801, USA; 4 Department of Psychology, Wayne State University, Detroit, MI 48202, USA; 5 Institute of Gerontology, Wayne State University, Detroit, MI 48202, USA

**Keywords:** splenium, genu, white matter, Rorschach Inkblot Method

## Abstract

Ideational slippage—characterized by incorrect word usage and strained logic during dialogue—is common in aging and, at greater frequency, is an indicator of pre-clinical cognitive decline. Performance-based assessment of ideational slippage may be useful in the study of cognitive aging and Alzheimer’s-disease-related pathology. In this preliminary study, we examine the association between corpus callosum volume and a performance-based assessment of ideational slippage in middle-aged and older adults (age 61–79 years). Ideational slippage was indexed from cognitive special scores using the Rorschach Inkblot Method (RIM), which are validated indices of deviant verbalization and logical inaccuracy (*Sum6*, *WSum6*). Among middle-aged and older adults, smaller splenium volume was associated with greater ideational slippage (η_p_^2^ = 0.48), independent of processing speed and fluid intelligence. The observed negative associations are consistent with visuospatial perception and cognitive functions of the splenium. The effect was strongest with the splenium, and volumes of the genu and total white matter had small effects that were not statistically significant. Conclusions: Results are discussed with future application of RIM special scores for the assessment of pre-clinical cognitive decline and, based on observed effect sizes, power analyses are reported to inform future study planning.

## 1. Introduction

The risk for Alzheimer’s disease and related dementia (ADRD) begins with incipient neurological pathology in middle-aged people [1], and early detection of pre-clinical cognitive decline is important for clinical research and practice. Typical ADRD symptoms include declarative memory impairment, slowed cognitive processing, and diminished working memory capacity [2]; these cumulative deficits may present in daily life as difficulties with organizing complex ideas and communicating them with accurate, contextual-embedded detail [3]. The use of the wrong word, logical inaccuracies, and other ideational slippage in the course of dialogue are often the first features of cognitive decline noticed by the individual and their family members [4]. An assessment of ideational slippage may, therefore, be useful for early detection of neurodegeneration and the study of ADRD-related pathology. In this preliminary report, we apply a standardized, performance-based assessment to the study of ideational slippage and its neural correlates in middle-aged and older adults.

In daily life, older adults display ideational slippage during complex, cognitively-demanding, and often ambiguous tasks [5]. Myriad assessments of cognitive and language function emphasize accuracy for a specific response [6,7], but few allow for unrestricted responses that can be evaluated for the quality and accuracy of thought and its verbal communication. One example of such a task is the Rorschach Inkblot Method (RIM), which provides an unrestricted opportunity to assess spontaneous language and reasoning as the individual interprets ambiguous stimuli based upon standardized procedures. The RIM is well suited for such study as it is a validated assessment of personality and cognitive function [8] and includes a wealth of normative data [9,10,11]. The RIM has a lengthy history of use in the study of cognitive aging [12,13,14,15], including in large-scale studies with modern administration and scoring systems [16,17]. Even when accounting for individual differences in fluid cognitive ability, older age is associated with less cognitive flexibility in RIM responses [18], although not all cross-sectional research has replicated this finding [17]. Longitudinal studies have the benefit of measuring age-related change without the confound of individual differences: such studies suggest increasingly restricted responses to the RIM among institutionalized adults age 70–100 years [19], and among nursing home residents (average age 76.4 year) [20]. Recent applications to patient populations have shown response differences between cognitive-typical older adults and patients with ADRD in special scores (*Sum6*, *Wsum6*) indicative of ideational slippage and, more broadly, cognitive impairment [21,22].

Six special scores on the RIM help to capture clarity of thinking when interpreting the measure’s ambiguous stimuli [9]. The *Sum6* summary score is the number of occurrences of ideational slippage during responses. This scale alone is insufficient, however, as deviations from logical and coherent thinking can range from relatively mild to relatively severe. For example, a response referring to an explorer trying to “circumvent the globe” is a benign error in word choice and a listener may take note of it but otherwise still understand the meaning of the speaker. In contrast, the statement “an explorer swishing the seven seas” is a more severe deviation that may indicate significant cognitive dysfunction, especially if the speaker did not notice the error. The *WSum6* index is a sum that gives greater weight to severe deviations and illogical responses. In this way, differentiations can be made between temporary falters in logic and persistent conceptual/judgment errors that are combined with strained reasoning resulting in lower cognitive clarity. Elevations in *Sum6* and *WSum6* primarily from benign deviations are hypothesized to occur in typical aging, consistent with decline in mental faculties that cause thoughts to be disorganized or incomplete [21,22]. Although a number of studies have examined age-related differences in RIM response number and quality, there has been no previous report of the *Sum6* and *WSum6* scores in a representative community-based sample of aging adults. Further application of this method in the study of age-related neurodegeneration requires an understanding of neural correlates of *Sum6* and *WSum6* scores; however, studies of neural correlates of RIM responses are rare and none have evaluated responses in middle-aged and older adults [23].

Based upon the task structure, known functional specialization in the brain and its vulnerability in aging, we hypothesize the corpus callosum may be closely related to ideational slippage on the task. The corpus callosum functionally integrates neural signals between brain hemispheres, and thus facilitates higher-order, complex visuospatial perception and cognitive processing [24] that are expected to be relevant to RIM responses. Across clinical disorders that display ideational slippage, patients have microstructural differences in the corpus callosum as compared to healthy control [25]. The corpus callosum is functionally heterogeneous, and atrophy of the splenium is associated with diminished visuospatial perception [26], whereas genu atrophy is correlated with deficits in executive function [27] and processing speed [28]. Critically, both anterior and posterior corpus callosum are sensitive to aging [29], and greater decline in splenium is evident in pre-clinical ADRD [30] and its progression [31] as compared to healthy counterparts. Thus, identifying correlations between RIM response measures of ideational slippage and volumes of anterior and posterior corpus callosum can inform future applications of the method in the study of cognitive aging and ADRD.

We evaluate this hypothesis in a preliminary report on a unique study of community-dwelling adults who underwent MRI and neuropsychological assessment. We hypothesize that smaller splenium and genu volumes will correlate with ideational slippage as indicated by high *Sum6* and *WSum6* scores. As a further evaluation of the effect with the corpus callosum, and not general white matter degeneration, we expect there will be no evidence of total white matter volume as a correlate of responses. Because MRI studies of RIM responses require substantial resources and specialized skills, we also report estimates of effect size and power to inform study planning to address future research applications.

## 2. Materials and Methods

### 2.1. Participants

The study was open for recruitment and data collection from September 2017 to August 2018. Participants were recruited from another ongoing study of brain aging in a metropolitan region in Midwest United States, in which participants consented to be contacted for additional study opportunities. From this existing cohort, 45 people were contacted by email or telephone call and were provided information about the study. To be eligible for study, all participants had no history or current diagnosis of cardiovascular disease, neurological disorders, or diseases effecting the central nervous system; head injury with loss of consciousness; corrected to normal vision and hearing; spoke English fluently; had no contraindications to MRI; reported right hand dominance. Following initial eligibility screening, 20 people were enrolled in the study and provided written informed consent following procedures approved by the university Institutional Review Board. Participants received USD 10 per hour compensation for participation in the study. The final sample for analysis that had complete data included 16 community-dwelling, middle-aged and older adults (age = 61–79 years; *M* = 72.00, *SD* = 5.51). The sample was predominantly White adults (93.8%) and included an equal number of men and women (see Table 1 for sample demographic description). Participants were on average college-educated (range 13–21 years). Participants were screened for dementia, and average scores on the Mini-Mental State Exam indicate typical cognition (all scores ≥ 27; *M* = 29.06, *SD* = 0.93). Self-report depression symptoms on the Center for Epidemiology Depression Scale indicated participants were on average below criteria (*M* = 9.53, *SD* = 8.43), and three participants scored above criteria (>16).

### 2.2. Rorschach Inkblot Method Administration and Scoring

In the present study, Rorschach administration and scoring were conducted according to the standardized rules of Exner’s Rorschach Comprehensive System (RCS) [9]. The protocols were administered and scored by a doctoral-level clinician with several years of Rorschach training and experience. Following RCS procedure, *Sum6* and *WSum6* summary scores were calculated. Low scores on *WSum6* do not provide significant interpretive value, and the score itself has no upper limit. As such, thresholds have been developed to identify potentially noteworthy elevations. Following recommended thresholds, scores were categorized into three groups: six participants were categorized as low (*WSum6* ≤ 10), four as elevations identified by Exner [9] as potentially meaningful (i.e., *WSum6* > 10), and six as elevations identified by the Rorschach international norms [11] as potentially meaningful (i.e., *WSum6* > 19). Among the scored protocols, a random 25% were subjected to a blind review to establish scoring accuracy with an intraclass correlation coefficient (ICC(3) [32]), which exceeded 0.90 for all special scores; 0.74 has been previously reported to indicate excellent coding reliability [33].

### 2.3. Cognitive Assessment

In addition to the RIM, participants completed 1 h of standardized neuropsychological assessments, including tests of processing speed and fluid intelligence. Tests were administered according to standard protocol. The Trails Making Test Version A is an assessment of processing speed [34], in which a longer time required to complete the task indicates slower ability. Cattell’s Culture Fair Intelligence assessment (version 3-b) includes four subtests of non-verbal, logical and matrix reasoning [35]. Sum total correct responses across the four subtests (not normed for age) were included as a covariate in analyses to account for variability in fluid cognitive ability when testing the relation between regional corpus callosum volumes and RIM response.

### 2.4. MRI Protocol and Corpus Callosum Volumetry

MRI data were acquired with a Siemens Magnetom 3 T Prismafit MRI scanner using a 64-channel head coil located in Beckman Institute’s Biomedical Imaging Center at the University of Illinois Urbana-Champaign. A 3D high-resolution T1-weighted magnetization prepared gradient echo (MPRAGE) sequence was collected with the following parameters: 0.9 mm^3^ isotropic voxel; repetition time = 1900 ms; inversion time = 900 ms; echo time = 2.32 ms, with GRAPPA and an acceleration factor of 2. Additional structural, functional, and diffusion weighted scans were acquired but are not part of the present report.

Cortical reconstruction and volumetric segmentation were completed with the Freesurfer (v 5.3) image analysis suite, which is available for download online (http://surfer.nmr.mgh.harvard.edu/; last accessed 30 March 2020). Briefly, images were submitted to motion correction [36], removal of non-brain tissue using a hybrid watershed/surface deformation procedure [37], automated Talairach transformation, and segmentation of white matter and deep gray matter regions [38]. This was followed by tessellation of the gray–white matter boundary, automated topology correction [37], and surface deformation following intensity gradients to optimize boundary delineation between gray and white matter and cerebrospinal fluid [39]. Volumes of the corpus callosum anterior (genu) and posterior (splenium) regions, and total white matter volume, were extracted for further analysis. Regional brain volumes were corrected for individual intracranial volume via regression [40].

### 2.5. Statistical Analysis

Prior to hypothesis testing, continuous variables were screened for normality and linearity, and all data were screened for multivariate outliers. One case presented as a univariate outlier in regional corpus callosum volumes and the values were winsorized. Preliminary analysis of age-related differences in RIM responses and regional corpus callosum volumes were evaluated with bivariate correlations for continuous variables.

Hypotheses were tested within a regression framework. First, total *Sum6* score was treated as a continuous variable in a univariate general linear model, predicted by adjusted volumes of the genu and splenium, including processing speed and fluid intelligence scores as covariates. Volumes of the genu and splenium were correlated (r = 0.67, *p* < 0.01); therefore, volumes were mean-centered to alleviate bias from multicollinearity when the measures were included in the same model. Second, *WSum6* threshold coded score was treated as an ordinal scale (0–2), predicted by the same set of variables. Analyses were repeated to test the control comparison with total white matter volume (values divided by 1000 for scale), in which a negligible effect size was hypothesized. Parameters are reported with standard effect sizes and model R^2^ (or Nagelkerke pseudo R^2^ estimates for ordinal regressions), and 95% confidence intervals (CI), which if not overlapping zero, provide further evidence in support of the hypothesized effect. Significance tests were corrected for multiple comparisons with Bonferroni correction (*p* < 0.02). To aid future study planning, a power analysis [41] was conducted based on the observed effect sizes.

## 3. Results

### 3.1. Observed RIM Responses and Age-Related Differences

Within the reported sample, *Sum6* scores were in the range of 1–8 (*M* = 4.53, *SD* = 2.40) and *WSum6* scores were in the range of 3–28 (*M* = 15.56, *SD* = 7.76) and were coded as an ordinal scale based upon recommended thresholds (see Table 1). Age was negatively, but not significantly, correlated with *Sum6* (r = −0.19, *p* = 0.49), total *WSum6* (r = −0.24, *p* = 0.38), and fluid intelligence (r = −0.01, *p* = 0.96), as well as a non-significant positive correlation with trails making task time (r = 0.34, *p* = 0.20) indicating a slowed processing speed. No age-related differences were observed in regional corpus callosum volumes (r = 0.20 and 0.37, *p*’s ≥ 0.16, anterior and posterior, respectively). Following this set of observations, hypotheses were tested in the sample without age as a covariate. See Table 2 for bivariate correlations among study variables.

### 3.2. Smaller Splenium Volume Was Associated with Ideational Slippage

**Figure 1:**
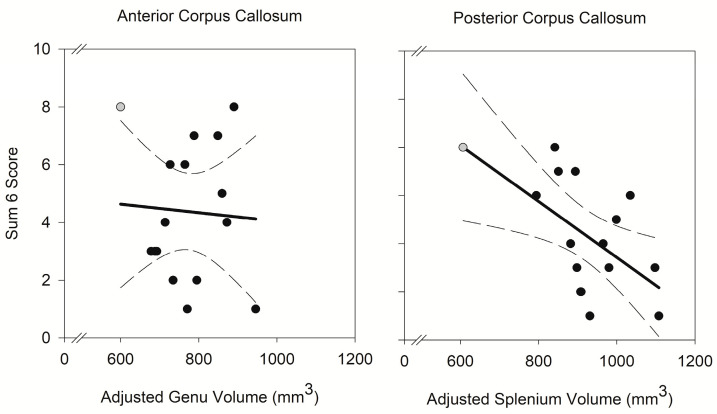
Correlation of regional corpus callosum volume with ideational slippage as indicated in RIM responses. Smaller splenium volume was associated with higher *Sum6* score (η_p_^2^ = 0.48, b = −0.05, *p* < 0.01) and genu volume was not (η_p_^2^ = 0.002, b = −0.003, *p* = 0.88). The regression relation is illustrated as a bold line, with 95% confidence intervals in broken lines. Regional volumes were adjusted for intracranial volume. The gray shaded data points represent winsorized values for a case that was identified as a univariate outlier.

In a multiple regression, smaller volume of the splenium (F (1, 11) = 10.10, *p* < 0.01), and not the genu (F (1, 11) = 0.03, *p* = 0.88), was associated with higher *Sum6* scores, independent of differences in processing speed (b = 0.05, F (1, 11) = 0.10, *p* = 0.75) and fluid intelligence (b = −0.74, F (1, 11) = 5.84, *p* = 0.03). The model explained approximately 55% of variance in responses (R^2^ = 0.55). The unique effect of splenium volume was moderate, η_p_^2^ = 0.48 (b = −0.05; 95% CI: −0.08/−0.01; Figure 1), and the non-significant unique effect of genu volume was small, η_p_^2^ = 0.002 (b = −0.003; 95% CI: −0.05/0.04).

Splenium volume also differentiated between *WSum6* levels defined by recommended thresholds. In an ordinal regression, smaller splenium volume predicted *WSum6* levels (b = −0.02, χ^2^ (1) = 5.33, *p* = 0.02; 95% CI: −0.03/−0.002), whereas genu volume (b = 0.01, χ^2^ (1) = 0.48, *p* = 0.49; 95% CI: −0.01/0.02), processing speed (b = −0.02, χ^2^ (1) = 0.12, *p* = 0.73; 95% CI: −0.15/0.11) and fluid intelligence (b = −0.22, χ^2^ (1) = 2.58, *p* = 0.11; 95% CI: −0.49/0.05) did not. The model explained a moderate amount of variance in the ordinal response scale (pseudo R^2^ = 0.47; χ^2^ (4, N = 16) = 8.59, *p* = 0.07).

Repeating analyses with total white matter volume, there was no evidence that the reported effect in splenium is due to a non-specific effect of white matter degeneration. Total white matter volume was unrelated to *Sum6* (b = −0.08, F (1, 12) = 0.62, *p* = 0.45; 95% CI: −0.31/0.15; η_p_^2^ = 0.05) and *WSum6* level (b = −0.03, χ^2^ (1) = 1.10, *p* = 0.29; 95% CI: −0.08/0.02).

### 3.3. Effect Size and Power Analysis for Future Study Planning

The observed effect of splenium volume in predicting *Sum6* scores was large (η_p_^2^ = 0.48, observed power = 0.70) and was supported by 95% CI, whereas the effect of genu volume was small (η_p_^2^ = 0.002, observed power = 0.05) and the 95% CI overlapped with zero. Based on the observed effect sizes, a sample N = 19–23 would achieve 80–90% power to detect the large effect of the splenium to significance (α = 0.05). However, a sample N = 125 would provide 80% power to detect a small effect (η_p_^2^ = 0.09) to significance, improve the 95% CI of the parameter estimate, and increase confidence when interpreting a null hypothesis test compared across regions.

## 4. Discussion

In a preliminary study of community-dwelling middle-aged and older adults who completed the RIM and underwent MRI, the association between ideational slippage and corpus callosum volume was assessed. Independent of fluid intelligence and processing speed, smaller splenium volume was associated with greater *Sum6* and *WSum6*, whereas correlations with volume of the genu were small and not statistically significant. Differential decline of the splenium is observed in aging and ADRD [30], and is associated with deficits in visuospatial perception [26]. Here, we provide evidence that a performance-based assessment of ideational slippage with the RIM is sensitive to variability in splenium volume among community-dwelling, middle-aged and older adults.

Both *Sum6* and *WSum6* provide an assessment of the respondent’s clarity of thinking. Among clinical populations, it has proven useful in the detection of formal thought disorders [9] as well as less severe pathologies [42]. Among nonclinical populations with mostly benign special scores, as reported here, elevations are more likely to reflect ideational slippage and not overt psychopathology *per se*. Indeed, the range of *Sum6* and *WSum6* scores was comparable to previous reports of a similar sample of community-dwelling, well-educated older adults [17].

The association of *Sum6* and *WSum6* with splenium (and smaller, not statistically significant effects of genu and total white matter volume) may indicate the vulnerability of RIM responses to disrupted visuospatial perception and reasoning in the course of aging. The splenium in the posterior corpus callosum is functionally connected with temporal and parietal regions involved in the integration and transfer of high-level sensory information [27], and contributes to visual figure–ground separation by integrating signal from the primary and secondary visual cortical areas [24]. These perceptual and cognitive functions are presumed necessary to generate responses to the ambiguous RIM stimuli and, critically, are vulnerable to decline in aging and related dementia. No prior study has identified neural correlates of RIM responses in the course of aging; however, those of RIM stimuli shown to healthy, young adults have identified correlates to cortical regions that are functionally connected to the splenium. For example, greater electroencephalogram activations in frontal parietal cortices were observed as participants considered the meaning of a monochromatic inkblot as compared to a polygon shape; it is noteworthy that somatosensory cortical activation was also observed for both stimulus conditions [43]. In functional near-infrared spectroscopy studies, functional modulation near the parietal lobe [44] and bilateral activity in the rostral frontal cortex [45] were reported among college-aged adults observing inkblots. Therefore, it is plausible that perceptual and fluid cognitive functions of the splenium support the creation and communication of RIM responses as indexed by *Sum6* and *WSum6*.

The corpus callosum is vulnerable to atrophy and microstructural changes in the course of typical aging [29] and assessment of ideational slippage may be useful for the study of pre-clinical cognitive decline. Pathology associated with ADRD appears to effect the splenium early in the disease [46] and shrinkage correlates with the severity of cognitive deficits and disease progression [31]. As prior reports have suggested *Sum6* and *WSum6* response scores may be sensitive to cognitive deficits that occur in ADRD [21,22], the observation of strong correlations of these scores with splenium volume among middle-aged and older adults without ADRD is intriguing. This preliminary report warrants additional study with longitudinal assessment to determine if RIM responses would be sensitive to cognitive declines indicative of pre-clinical ADRD.

The study should be interpreted with consideration of its strengths and limitations. Here we report on a novel sample of community-dwelling, middle-aged and older adults who completed the RIM and underwent structural MRI. The sample is small and includes high-functioning, well-educated adults who were predominantly White. Based upon the observed effect sizes and reported power analyses, future studies should endeavor to recruit larger, more diverse samples that can improve the generalizability of the findings to the world’s population. The hypothesis tested here would benefit from replication and extension to individuals with clinical cognitive declines. Moreover, we tested a specific hypothesis pertaining to the corpus callosum subregions; additional brain regions and cognitive functions are expected to be relevant to generate RIM responses, which should be explored further in a larger study. Finally, this report is a cross-sectional study of individual differences in regional brain volumes as correlates of RIM responses, and as such, no causal inferences or determination of mechanisms of neurodegeneration can be made. Future studies should build upon this preliminary report to include prospective longitudinal study of individuals with pre-clinical and clinical ADRD to evaluate the sensitivity of RIM responses to disease onset and progression.

## 5. Conclusions

In a novel sample of community-dwelling, middle-aged and older adults, we identify white matter neural correlates of ideational slippage as measured in RIM responses. High *Sum6* and *WSum6* scores correlated with smaller splenium volumes, independent of processing speed and fluid intelligence. The effect was strongest with splenium volumes, and the independent effects of genu and total white matter volumes were small and not statistically significant. Taken together, the RIM may be sensitive to ideational slippage that correlates with white matter deterioration that is common in aging and ADRD. We speculate that performance-based measures, such as the RIM, may be sensitive to detect pre-clinical neural and cognitive declines, and future application should consider if it is sensitive to identify persons at risk to develop ADRD.

## Figures and Tables

**Table 1:** Description of sample demographic variables and RIM summary scores.

Variable	Total Sample	Women	Men
Sample Size	16	8	8
Age (years; Mean ± SD)	72.00 ± 5.51	71.63 ± 5.37	72.38 ± 6.00
% female	50.00%		
Education (years; Mean ± SD)	17.08 ± 2.39	15.71 ± 1.70	19.00 ± 1.87
MMSE (Mean ± SD)	29.06 ± 0.93	28.88 ± 1.13	29.25 ± 0.71
CES-D (Mean ± SD)	9.53 ± 8.43	10.88 ± 10.72	8.00 ± 5.16
*Sum6* (Mean ± SD)	4.38 ± 2.39	4.88 ± 2.03	3.88 ± 2.75
*WSum6 Total* (Mean ± SD)	15.56 ± 7.76	17.13 ± 6.51	14.00 ± 9.01
*WSum6 Coded* (Median, IQR)	1.00, IQR = 2.00	1.00, IQR = 2.00	1.00, IQR = 2.00

Note: Sample demographic variables are reported with mean and standard deviation (SD), except for *WSum6 Coded* score, which was an ordinal scale and is reported with median and interquartile range (IQR). MMSE—Mini-Mental State Exam; CES-D—Center for Epidemiology Depression Scale; *Sum6* and *WSum6* summary special scores from the Rorschach Inkblot Method (RIM). *WSum6* scores are reported as observed total score and coded scores for interpretative threshold.

**Table 2:** Bivariate correlations among study variables.

	1	2	3	4	5	6
1. Age						
2. *Sum6*	−0.19					
3. *WSum6 Total*	−0.24	0.96 *				
4. Fluid Cognitive Ability	−0.01	−0.17	−0.32			
5. Processing Speed	0.34	−0.10	−0.09	−0.20		
6. Anterior Corpus Callosum Volume	0.20	−0.06	−0.09	−0.23	0.58 *	
7. Posterior Corpus Callosum Volume	0.37	−0.59 *	−0.54 *	−0.30	0.35	0.30

Note: Pearson *r* correlations are reported for study variables measured on continuous scales. * indicates statistical significance (*p* < 0.05).

## Data Availability

The data presented in this study are available on request from the corresponding author due to privacy protection.
